# Brain effects of gestating germ-free persist in mouse neonates despite acquisition of a microbiota at birth

**DOI:** 10.3389/fnins.2023.1130347

**Published:** 2023-05-03

**Authors:** Alexandra Castillo-Ruiz, Aviva Gars, Hannah Sturgeon, Nicole M. Ronczkowski, Dhanya N. Pyaram, Charlène J. G. Dauriat, Benoit Chassaing, Nancy G. Forger

**Affiliations:** ^1^Neuroscience Institute, Georgia State University, Atlanta, GA, United States; ^2^Medical College of Georgia, Augusta University, Augusta, GA, United States; ^3^INSERM U1016, Team “Mucosal Microbiota in Chronic Inflammatory Diseases,” Université Paris Cité, Paris, France

**Keywords:** cross-fostering, cell death, microglia, forebrain size, bacterial load, colonic content

## Abstract

At birth, mammals experience a massive colonization by microorganisms. We previously reported that newborn mice gestated and born germ-free (GF) have increased microglial labeling and alterations in developmental neuronal cell death in the hippocampus and hypothalamus, as well as greater forebrain volume and body weight when compared to conventionally colonized (CC) mice. To test whether these effects are solely due to differences in postnatal microbial exposure, or instead may be programmed *in utero*, we cross-fostered GF newborns immediately after birth to CC dams (GF→CC) and compared them to offspring fostered within the same microbiota status (CC→CC, GF→GF). Because key developmental events (including microglial colonization and neuronal cell death) shape the brain during the first postnatal week, we collected brains on postnatal day (P) 7. To track gut bacterial colonization, colonic content was also collected and subjected to 16S rRNA qPCR and Illumina sequencing. In the brains of GF→GF mice, we replicated most of the effects seen previously in GF mice. Interestingly, the GF brain phenotype persisted in GF→CC offspring for almost all measures. In contrast, total bacterial load did not differ between the CC→CC and GF→CC groups on P7, and bacterial community composition was also very similar, with a few exceptions. Thus, GF→CC offspring had altered brain development during at least the first 7 days after birth despite a largely normal microbiota. This suggests that prenatal influences of gestating in an altered microbial environment programs neonatal brain development.

## 1. Introduction

Microbiota from maternal and environmental sources rapidly colonize all epithelial surfaces of mammalian neonates at birth. Disruptions of the maternal microbiota during pregnancy, such as those resulting from a high fat diet or antibiotic treatment, alter the vertical transmission of microbes from mother to offspring and have long-term effects on offspring physiology and behavior (Olszak et al., 2012; Bokulich et al., 2016; Leclercq et al., 2017; Schulfer et al., 2018; Chen et al., 2021a,b; O’Connor et al., 2021). In addition, several recent studies suggest *in utero* effects of the maternal microbiota on fetal development (Humann et al., 2016; Tochitani et al., 2016; Kim et al., 2017; Thion et al., 2018; Pronovost and Hsiao, 2019; Vuong et al., 2020), due to the presence of bacterial metabolites in maternal circulation that cross the placenta or other signaling mechanisms.

By far the largest population of microbes resides in the distal gastrointestinal tract (i.e., the colon), with bacteria comprising the vast majority of those microorganisms (Sender et al., 2016). The gut microbiota communicates reciprocally with the brain via the gut-microbiota-brain axis (Cryan and Dinan, 2012; Morais et al., 2021), and animals living in the absence of microbes [i.e., germ-free (GF)] have played a crucial role in establishing this link. GF mice have an altered neuroendocrine stress response, changes in hippocampal neurogenesis, reduced anxiety, and altered social behavior in adulthood compared to conventionally colonized (CC) controls (e.g., Sudo et al., 2004; Diaz Heijtz et al., 2011; Clarke et al., 2013; Ogbonnaya et al., 2015). Some of these changes are normalized by introducing a microbiota in adulthood or adolescence, but others persist, suggesting early life neural programming. However, the specific brain processes affected early in life by microbe exposure are largely unknown.

Microglia are the macrophages and primary innate immune cells of the brain, and they respond to the microbiota throughout life. GF adults have increased microglial numbers but decreased microglial responsiveness to immune challenges compared to controls (Erny et al., 2015; Matcovitch-Natan et al., 2016). The co-housing of GF dams and their litters with CC female mice soon after birth reduces microglial numbers in comparison to GF mice when examined in adulthood (Erny et al., 2021), suggesting a normalization of microglia in GF mice by long-term postnatal colonization. How quickly the normalization occurs, however, is unknown. This is an important question because current obstetric practices routinely alter the microbiota of pregnant mothers and their babies. For example, 40% of mothers in the United States are treated peripartum with antibiotics (Ledger and Blaser, 2013; Martinez de Tejada, 2014) that cause a marked depletion of their microbiota and that of their offspring. Even transient alterations in the microbiota during perinatal life could have lasting effects on offspring brain development, given the many important neurodevelopmental events that occur during the early postnatal period. In rodents, a depletion of the maternal/prenatal or postnatal microbiota by antibiotics alters social behaviors and anxiety-like behavior in the offspring in adolescence and adulthood (Tochitani et al., 2016; Leclercq et al., 2017; O’Connor et al., 2021; Lynch et al., 2023).

Microglial colonization and naturally occurring cell death are two of the most salient neurodevelopmental events occurring around the time of birth in mice. We recently showed that, compared to CC mice, those that are gestated and born into a GF environment have increased microglial labeling and altered neuronal cell death in the brain during the newborn period (Castillo-Ruiz et al., 2018a). It is unknown whether these changes are due solely due to the postnatal absence of microbes, or whether the maternal microbiota may program offspring brain development before birth. To test this, mice in the current study were gestated and born to a GF mother and then cross-fostered immediately after birth to CC dams; newborns fostered within microbial status served as controls. Colon contents and brains of offspring were collected 7 days later to compare bacterial colonization of the gut and several measures of brain development. Our results suggest that maternal microbial status *in utero* has a prolonged effect on neonatal brain development.

## 2. Materials and methods

### 2.1. Animals

Adult Swiss Webster GF and CC mice were purchased from Taconic Biosciences (Germantown, NY, USA). All mice were housed in our GF facility in an isolated, ventilated caging system (Isocage, Techniplast, Buguggiate VA, Italy). Mice were maintained on a 12:12 light-dark cycle with *ad libitum* access to autoclaved food and water. All animal procedures were approved by Georgia State University’s Institutional Animal Care and Use Committee (protocol #A20013) and followed the National Institutes of Health Guide for the Care and Use of Laboratory Animals.

### 2.2. Cross-fostering procedure

**FIGURE 1:**
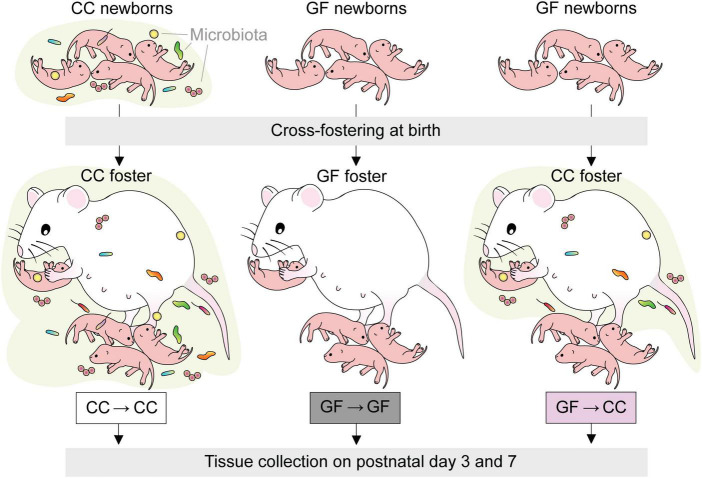
Experimental design. GF newborns were cross-fostered immediately after birth to CC dams (GF→CC group) and compared to offspring fostered within the same microbiota status (CC→CC, GF→GF groups).

Females and males were housed together for 1–4 days. Beginning on the eve of the first possible embryonic day (E) 19, we performed hourly, around-the-clock checks for births, with checks during the dark period performed under red light illumination. Immediately upon observing the birth of a litter, cages were thoroughly sprayed with a sterilizing solution (1 part Exspor base: 1 part Expsor activator: 4 parts tap water; Ecolab Inc., Saint Paul, MN, USA) and placed within a biosafety cabinet that prior to the procedure had been UV treated and sprayed with the sterilizing solution. Offspring were gently transferred to a sterile container using a sterile set of tweezers before being assigned to a foster dam that had given birth within the previous 48 h. The foster dam’s own pups were removed and experimental pups (whole litters) were then placed in the foster dam’s cage under sterile conditions. We cross-fostered GF pups to CC dams (GF→CC group; *n* = 34), and, to control for the cross-fostering procedure, CC and GF pups to dams within the same microbiota status (CC→CC group, *n* = 37 and GF→GF group, *n* = 15) (Figure 1). In two additional cases, foster mothers were not available for control litters (one CC→CC *n* = 17 and one GF→GF litter *n* = 10) and these pups were sham cross-fostered; that is, they underwent all the procedural steps of cross-fostering (spraying of cages, placement of pups in sterile holding container) but pups were returned after a delay to the birth mother. Sham cross-fostered mice did not differ from pups fostered to an unrelated mother for any dependent variable tested (determined by ANOVA or t-tests within microbial status, as appropriate) and are therefore included in the analyses below and identified as sham cross-fostered on all figures. The total number of litters represented in each group was four for CC→CC, two for GF→GF and three for GF→CC. Note that due to low GF pregnancy rates, it was challenging to foster GF pups within microbial status; this explains the lower number of litters and subjects for the GF→GF group.

### 2.3. Tissue collection

To assess how rapidly gut colonization takes place, we sacrificed half of each litter at P3 and collected colon contents from a subset of mice (CC→CC *n* = 16; GF→CC: *n* = 12; GF→GF *n* = 14). To assess brain effects related to bacterial colonization of the gut, we collected brains (CC→CC *n* = 20; GF→CC: *n* = 12; GF→GF *n* = 10) and colon contents (CC→CC *n* = 17; GF→CC: *n* = 10; GF→GF *n* = 10) of a subset of offspring at P7. On collection days, mice were weighed and immediately euthanized via rapid decapitation 8–10 h after lights on. Brains (P7) were fixed in 5% acrolein in 0.1 M phosphate buffer for 24 h at room temperature and then transferred to 30% sucrose at 4°C, followed by cryoprotection at −20°C until sectioning. Colon contents (P3 and P7) were collected by excising the colon and gently extruding contents with the flat surface of a curved, sterile tweezer. Contents were weighed, and stored at −80°C prior to processing.

### 2.4. Immunohistochemistry

Brains were coronally sectioned on a freezing microtome into four, 40 μm series. Sections were collected into cryoprotectant solution and stored at −20°C. One series was processed for the immunohistochemical detection of ionized calcium binding adaptor molecule 1 (Iba1) to label microglia, and two series for the detection of activated caspase-3 (AC3) to identify dying cells. Unless otherwise stated, tissue was washed between steps in 1X tris buffered saline (TBS) and all steps were carried out at room temperature. Epitope retrieval was performed with 0.05 M sodium citrate for 1 h for Iba1 or 30 min for AC3. Then, unreacted aldehyde was blocked via incubation with 0.1 M glycine for 30 min, followed by an incubation in a blocking solution (20% normal goat serum (NGS), 1% H_2_O_2_, 0.3% Triton X in TBS), and an overnight incubation with the primary antibody: rabbit anti-Iba1 (Wako, Chuo-Ku, Osaka, Japan; 1:3,000; 2% NGS, 0.3% Triton X in TBS) or rabbit anti-AC3 (Cell Signaling, Beverly, MA, USA; 1:5,000; 2% NGS, 0.3% Triton X in TBS). Sections were washed in a dilute blocking solution (1% NGS, 0.02% Triton X in TBS), incubated for 1 h in a goat anti-rabbit secondary antibody (Vector Laboratories, Burlingame, CA, USA; 1:1,000 for Iba1 or 1:500 for AC3; 0.32% Triton X in TBS), washed in 1X TBS-0.2% Triton X, and incubated for 1 h in an avidin–biotin solution (Vector Laboratories; 1:1,000 for Iba1 or 1:500 for AC3 in 1X TBS). Tissue was washed in acetate buffer and incubated in 0.02% diaminobenzidine tetrahydrochloride, 2% nickel sulfate, and 0.0025% H_2_O_2_ made in the same buffer. Sections were mounted onto gelatin-coated slides, counterstained with thionin in the case of AC3-immunoreacted tissue, dehydrated, and coverslipped.

### 2.5. Quantification of microglia, dying cells, and forebrain size

All analyses were performed on coded slides by an investigator blind to treatment group. We analyzed brain regions where we previously observed differences between neonatal GF and CC mice: the paraventricular nucleus of the hypothalamus (PVN), the CA1 oriens layer of the hippocampus, and the arcuate nucleus (ARC) (Castillo-Ruiz et al., 2018a). In addition, we included the primary somatosensory cortex (S1) in our analyses of microglia as microbiota-dependent effects have been previously reported for microglia in this region (Thion et al., 2018). For the PVN, we analyzed all available sections, starting when the nucleus has a tubular shape (Plates 127-131 in Paxinos et al., 2007). For the CA1 oriens, we included sections from the rostral-most appearance of the dentate gyrus (Plate 128) to the point where the hippocampus starts to tip ventrally (Plate 131). For the ARC, sampling started at the point where the nucleus has a well-defined triangular shape (Plate 133) and ended when the nucleus was no longer visible (Plate 142). S1 was analyzed in three consecutive sections, starting where the dentate gyrus is clearly defined (Plate 128) and ending when the hippocampus tips ventrally (Plate 131), as described in Strahan et al. (2017).

Slides were scanned using a Hamamatsu Nanozoomer (Hamamatsu Photonics K. K. Hamamatsu City, Japan) and cell quantification was performed using Aperio Image Scope (Leica Biosystems Inc., Buffalo Grove, IL, USA). Contours were drawn around the regions of interest and the number of microglia and dying cells within those contours was recorded. The sum of AC3+ and Iba1+ counted cells across all sections in each animal was divided by total area sampled, and then multiplied by section thickness to obtain cell density per mm^3^.

To assess forebrain size, we outlined the left side of the forebrain in one series of the AC3 labeled tissue, using six alternate sections, starting from the section where the medial border of the anterior commissure lies ventral to the tip of the lateral ventricle (Plate 117) and ending at the section with the rostral most appearance of the dorsomedial nucleus of the hypothalamus (Plate 133), as previously described (Castillo-Ruiz et al., 2018a). The sum of areas across all sections was multiplied by two and then by section thickness to obtain overall forebrain volume in mm^3^ for each animal.

### 2.6. DNA extraction from colon contents

Deoxyribonucleic acid extraction from colon contents was performed using the QIAamp fast DNA stool mini kit (Qiagen LLC, Germantown, MD, USA) according to the manufacturer’s instructions, with the addition of a bead beating step at the beginning of the procedure to aid with homogenization: samples were transferred to PowerBead Pro Tubes (Qiagen) and agitated for 2 min in the Mini-Beadbeater (Biospec Products, Inc., Bartlesville, OK, USA). The stock DNA was used for polymerase chain reaction (PCR) and sequencing analysis of the 16S rRNA gene.

### 2.7. 16S rRNA PCR for total bacterial load

Polymerase chain reaction was performed in the C1000 Touch Thermal Cycler (Bio-Rad, Hercules, CA, USA) (2 min at 95°C, followed by 40 cycles of 5 s at 95°C and 10 s at 60°C) using a QuantiNova SYBR green PCR kit (Qiagen) with universal 16S rRNA primers 8F: 5′-AGAGTTTGATCCTGGCTCAG-3′ and 338R: 5′-CTGCTGCCTCCCGTAGGAGT-3′. Negative controls were run concurrently and included clean paper towels used for sample collection and buffer from the DNA extraction kit. The quantitative cycle (Cq) values for negative control and GF samples were very close to the final cycle of the PCR run (mean = 38.47; SEM = 0.19; compare these values with the much earlier read outs from CC groups: mean = 22.51; SEM = 0.31). In order to calculate fold-increase in bacterial load in CC groups, we used the GF Cq values as reference. Bacterial load was calculated using the formula 2^–(Δ*Cq*)^, where ΔCq was obtained by subtracting the Cq average of the GF→GF group from each individual animal’s Cq value. Fold-change values were then obtained by dividing each experimental value by the average for the GF→GF group.

### 2.8. 16S rRNA gene sequencing and analysis

16S rRNA gene amplification and sequencing were performed using Illumina MiSeq technology (Illumina Inc., San Diego, CA, USA). The 16S rRNA genes, region V4, were PCR amplified from each sample using a composite forward primer and a reverse primer containing a unique 12-base barcode, designed using the Golay error-correcting scheme, which was used to tag PCR products from respective samples (Caporaso et al., 2012). We used the forward primer 515F 5′- *AATGATACGGCGACCACCGAGATCTACACGCT*XXXXXXXXXXXX**TATGGTAATT*GT***GTGYCAGCMGCCGCGGTAA-3′: the italicized sequence is the 5′ Illumina adaptor, the 12 X sequence is the Golay barcode, the bold sequence is the primer pad, the italicized and bold sequence is the primer linker, and the underlined sequence is the conserved bacterial primer 515F. The reverse primer 806R used was 5′-*CAAGCAGAAGACGGCATACGAGAT***AGTCAGCCAG*CC***GGACTACNVGGGTWTCTAAT-3′: the italicized sequence is the 3′ reverse complement sequence of Illumina adaptor, the bold sequence is the primer pad, the italicized and bold sequence is the primer linker and the underlined sequence is the conserved bacterial primer 806R. PCR was performed using a Hot Master PCR mix (Quantabio, Beverly, MA, USA) in the C1000 Touch Thermal Cycler (3 min at 95°C, followed by 30 cycles of 45 s at 95°C, 60 s at 50°C and 90 s at 72°C). PCR products were purified with Ampure magnetic purification beads (Agencourt, Brea, CA, USA), and visualized by gel electrophoresis. Products were then quantified (BioTek Fluorescence Spectrophotometer; BioTek Instruments, SAS, France) using Quant-iT PicoGreen dsDNA assay (Invitrogen, Carlsbad, CA, USA). A master DNA pool was generated from the purified products in equimolar ratios. The pooled products were quantified using Quant-iT PicoGreen dsDNA assay and then sequenced using an Illumina MiSeq sequencer (paired-end reads, 2 × 250 bp) at Cornell University, Ithaca.

Sequences were demultiplexed and quality filtered using the Dada2 method (Callahan et al., 2016) with QIIME2 default parameters in order to detect and correct Illumina amplicon sequence data, and a table of QIIME2 artifact was generated. A tree was next generated, using the QIIME fragment-insertion sepp command, for phylogenetic diversity analyses, and alpha and beta diversity analyses were computed using the core-metrics-phylogenetic command. For taxonomy analysis, features were assigned to amplicon sequence variants (ASVs) with a 99% threshold of pairwise identity to the Greengenes reference database 13_8 (McDonald et al., 2012).

### 2.9. Statistics

We combined the data for males and females in all analyses below. Preliminary analyses did not identify significant effects of sex for any variable, although some comparisons may have been under-powered for identifying sex differences. One-way ANOVA was used to evaluate cross-fostering effects on microglial number, cell death, body weight, forebrain size, colon content weight, and bacterial diversity. When applicable, ANOVA was followed by Fisher’s least significant difference. Non-parametric tests (Kruskal–Wallis followed by Dunn’s test) were performed for bacterial load as data did not conform to the homogeneity of variance assumption of ANOVA. Two-tailed independent samples *t*-tests were used to test the effects of cross-fostering on metrics of alpha diversity: ASVs and Shannon diversity index. Principal coordinate analysis (PCoA) plots of Bray Curtis distances were used to assess the variation between the experimental groups (beta diversity), which was further tested via Permutational analysis of variance (PERMANOVA). Analysis of composition of microbiomes (ANCOM) was used to identify differentially abundant species between groups, and one-tailed independent samples *t*-tests or Mann–Whitney tests were used to confirm differences. Statistical analyses were performed using GraphPad Prism (GraphPad software LLC, San Diego, CA, USA) and QIIME2 (Bolyen et al., 2019). Two immunohistochemical runs were performed per marker (Iba1, AC3), with half of the subjects per group included in each run. The second run for Iba1, however, was unsuccessful so animal numbers are lower for Iba1 than for AC3 analyses.

## 3. Results

### 3.1. Microglial effects of gestating germ-free persist in some brain regions despite introduction to a microbiota at birth

**FIGURE 2:**
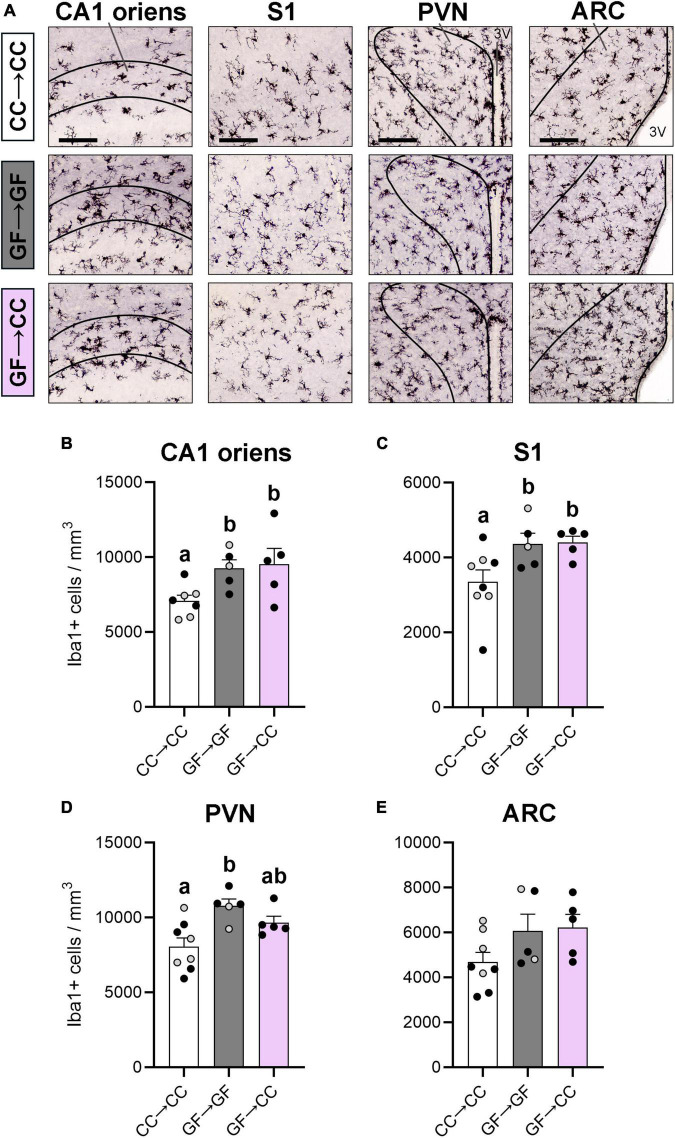
Microglial effects of gestating germ-free persist in mouse neonates despite introduction to a microbiota at birth. **(A)** Photomicrographs of Iba1 + stained tissue in representative CC→CC, GF→GF, and GF→CC mice, showing the brain regions analyzed: CA1 oriens, S1, PVN, and ARC (regions smaller than field of view indicated with black lines). 3V, third ventricle. Scale bar = 100 μm. **(B,C)** Microglial density was higher in groups gestated GF in the CA1 oriens **(B)** and S1 **(C)**, regardless of introduction to a microbiota at birth in the GF→CC group. **(D)** In contrast, microglial density in the PVN was no different between GF→CC and either control group, suggesting partial normalization of the microglial phenotype by microbiota introduction at birth. **(E)** No differences between groups were seen in the ARC. Group means with different letters are significantly different from each other. Mean + SEM and individual data points are depicted, with gray symbols representing sham cross-fostered mice in control groups.

We first examined microglia in four brain regions in which we or others have reported effects of GF status. Specifically, microglial labeling is increased in the PVN, ARC, cortex, and CA1 oriens layer of the hippocampus in perinatal or adult GF mice (Erny et al., 2015; Castillo-Ruiz et al., 2018a; Thion et al., 2018). Here, we found significant effects of group in the CA1 oriens [*F*(2,14) = 4.40, *p* = 0.03], S1 [*F*(2,15) = 4.40, *p* = 0.03], and PVN [*F*(2,15) = 6.77, *p* = 0.008] (Figures 2A–D). As seen previously when comparing GF and CC mice, GF→GF mice had more microglia than CC→CC mice in these brain regions (*p*_*s*_ ≤ 0.03). Remarkably, the introduction of a microbiota at birth was not sufficient to change the GF phenotype in the CA1 oriens or S1, as microglial number in GF→CC mice remained significantly higher than in CC→CC mice (*p*_*s*_ ≤ 0.03) and was no different from GF→GF mice at P7 (Figures 2B, C). In contrast, the PVN showed partial normalization of microglial phenotype as the GF→CC group did not differ from either the GF→GF or CC→CC groups (Figure 2D). For the ARC, there was no difference between groups in the overall ANOVA [*F*(2,15) = 2.53, *p* = 0.11] (Figure 2E).

### 3.2. Cell death effects of gestating germ-free persist despite introduction to a microbiota at birth

**FIGURE 3:**
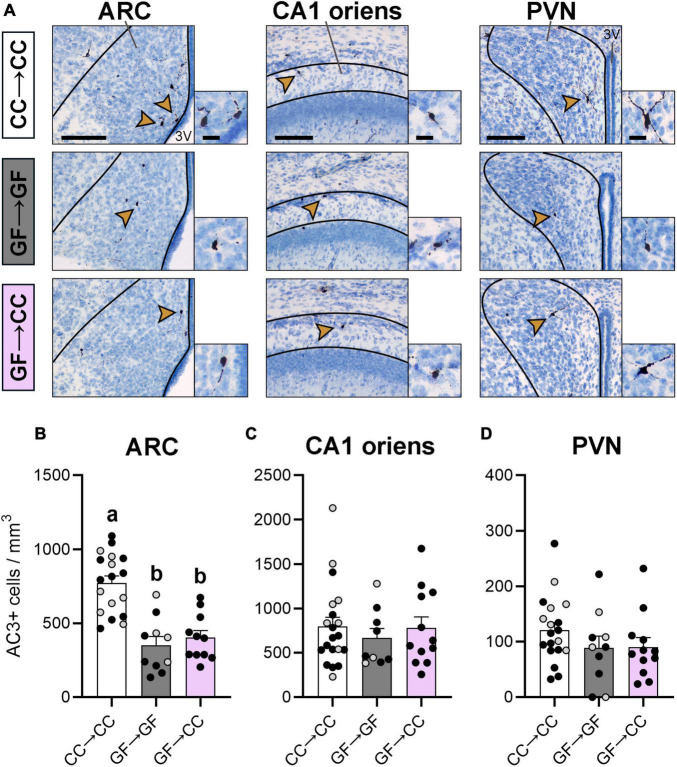
Cell death effects of gestating germ-free persist in the ARC of mouse neonates despite introduction to a microbiota at birth. **(A)** Photomicrographs of AC3 + stained tissue (counterstained with thionin) in representative CC→CC, GF→GF, and GF→CC mice, showing the brain regions analyzed: ARC, CA1 oriens, and PVN (all regions indicated with black lines). Arrowheads point to cells shown at higher magnification in the insets. 3V, third ventricle. Scale bar = 100 μm (main photomicrograph) and 20 μm (insets). **(B)** Cell death density was lower in groups gestated GF in the ARC, regardless of introduction to a microbiota at birth in the GF→CC group. **(C,D)** Cell death density did not differ between groups in the CA1 oriens **(C)** or PVN. **(D)** Group means with different letters are significantly different from each other. Mean + SEM and individual data points are depicted, with gray symbols representing sham cross-fostered mice in control groups.

Compared to CC mice, we previously observed increased cell death in the CA1 oriens and PVN and reduced cell death in the ARC of GF mice on P0 and P3 (Castillo-Ruiz et al., 2018a). Here, we again found an effect in the ARC [*F*(2,36) = 22.28, *p* < 0.0001] and, as before, the GF→GF group had fewer dying cells than the CC→CC group (Figures 3A, B). Importantly, introduction to a microbiota at birth was not sufficient to change this phenotype as the GF→CC group did not differ from the GF→GF group and remained different from the CC→CC group at P7 (*p* < 0.0001). We did not find an effect of group in the CA1 [*F*(2,38) = 0.30, *p* = 0.74] or PVN [*F*(2,38) = 1.33, *p* = 0.28], perhaps because P7 is well after the peak of cell death in these regions (Figures 3C, D; Mosley et al., 2017).

### 3.3. Gross measurement effects of gestating germ-free persist in mouse neonates despite introduction to a microbiota at birth

**FIGURE 4:**
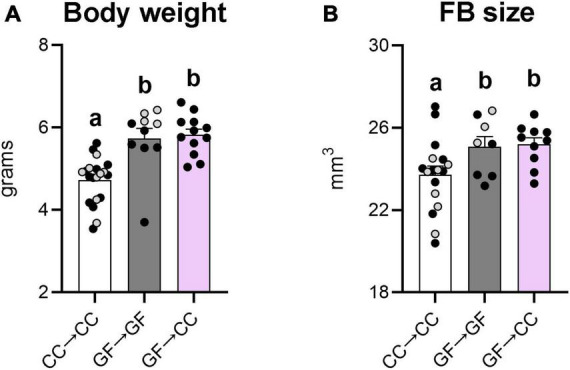
Effects of gestating germ-free on body weight and forebrain size persist in mouse neonates despite introduction to a microbiota at birth. Body weight **(A)** and forebrain size **(B)** were greater in GF→GF and GF→CC mice, in comparison to the CC→CC group. Group means with different letters are significantly different from each other. Mean + SEM and individual data points are depicted, with gray symbols representing sham cross-fostered mice in control groups.

Our previous study also showed greater body weight and forebrain size in GF neonates compared to CC controls (Castillo-Ruiz et al., 2018a). Here we again found significant effects of group for both measures [*F*(2,39) = 16.41, *p* < 0.0001 and *F*(2,32) = 3.89, *p* = 0.03, respectively], and similar to what we observed previously, the GF→GF group weighed more and had a larger overall forebrain size (Figures 4A, B) than the CC→CC group (*p*_*s*_ ≤ 0.04). The GF→CC mice remained significantly different from CC→CC mice (*p*_*s*_ ≤ 0.02) for both measures (Figures 4A, B) and were no different from GF→GF mice on either measure.

### 3.4. Cross-fostering largely normalizes gut bacterial load and composition

**FIGURE 5:**
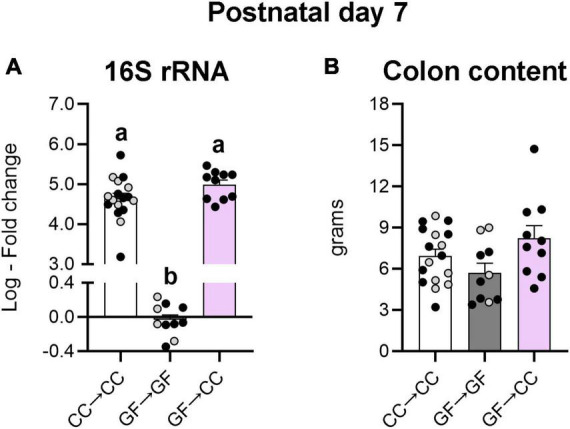
Introduction to a microbiota at birth normalizes the bacterial load of mice gestated germ-free at P7. **(A)** Relative quantification of the 16S rRNA gene from colon content showed similar levels of bacterial DNA in the groups harboring microbiota. The GF→GF group was used as reference group for fold change calculations. **(B)** Size of the colon content sample was unlikely to affect the assessment of bacterial load as there were no differences in this measure between groups. Group means with different letters are significantly different from each other. Mean + SEM and individual data points are depicted, with gray symbols representing sham cross-fostered mice in control groups.

Persistence of the GF phenotype seen above in the GF→CC group could be related to differences in the amount (load) and/or identity (composition) of gut microbial species. To test these hypotheses, we first assessed bacterial load in colon contents 7 days after birth. Not surprisingly, a non-parametric one-way ANOVA revealed significant effects of group on bacterial load (*H*_2_ = 23.38, *p* < 0.0001), with CC→CC and GF→CC groups having approximately 10^6^ -fold greater bacterial load than the GF→GF group (*p*_*s*_ ≤ 0.0002). Importantly, the CC→CC and GF→CC groups did not differ from each other on this measure (Figure 5A). This effect is unlikely driven by group differences in colon content size as there was no effect of group on this measure [*F*(2,34) = 2.84, *p* = 0.07] (Figure 5B).

**FIGURE 6:**
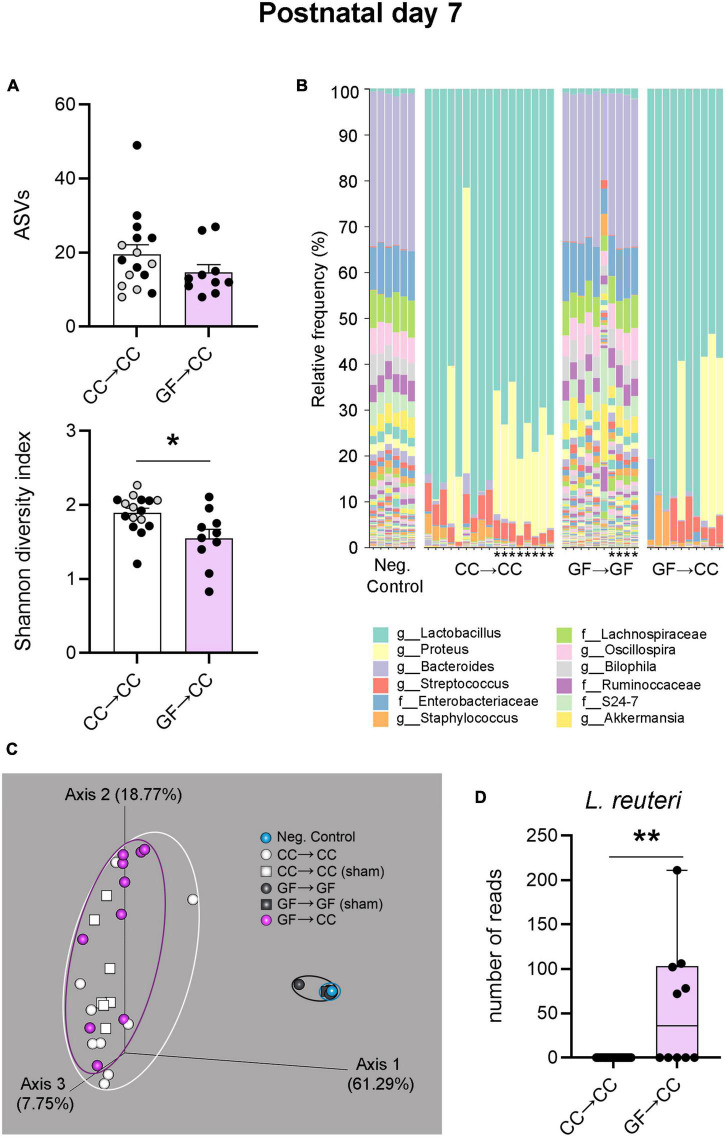
Introduction to a microbiota at birth largely normalizes bacterial composition of mice gestated germ-free by P7. **(A)** Measures of alpha-diversity revealed no difference between CC→CC and GF→CC groups in the number (richness) of ASVs (top). In contrast, when richness and abundance were considered by using the Shannon diversity index, the GF→CC group showed slightly lower diversity (bottom). Mean + SEM and individual data points are depicted, with gray symbols representing sham cross-fostered mice in control groups. *p = 0.01. **(B)** Relative abundance of bacterial groups per sample (columns), showing that overall bacterial composition was normalized in the GF→CC group as this group was similar to the CC→CC controls but markedly different from negative control samples and GF→GF controls. Asterisks identify the sham cross-fostered mice in control groups. The 12 most abundant taxa are shown in the color key. Sequences were classified to the lowest taxonomic level that could confidently be identified. f, family; g, genus. **(C)** PCoA plots based on Bray-Curtis dissimilarity, showing that GF→CC and CC→CC groups were similar in bacterial community composition as individual samples (symbols) clustered together but separate from controls (clustering indicated with ellipses). Note that most samples for negative control and GF→GF groups overlap due to tight clustering; *n* = 6 and 10 for those groups, respectively. Percent of variance explained by principal coordinates is indicated on the axes. **(D)** Boxplots of the number of reads per sample of the ASV identified as *Lactobacillus reuteri*. While the CC→CC group did not return positive *L. reuteri* reads, half of the samples in the GF→CC group did. **p = 0.003.

We next assessed bacterial composition through 16S rRNA gene sequencing. Metrics of alpha diversity showed that there was no difference in ASVs (richness) between GF→CC and CC→CC groups (Figure 6A, top). However, there was a difference between these groups when richness and abundance (evenness) were considered using the Shannon diversity index: the GF→CC group had slightly lower diversity [*t*(24) = 2.77, *p* = 0.01] (Figure 6A, bottom). Taxon abundance assessment revealed that the colonic microbiota of GF→CC and CC→CC groups were remarkably similar but vastly distinct from negative control samples. The presence of a bacterial signal in 16S rRNA amplification of negative control samples is expected, as it captures any environmental contamination as well as the so-called “kit-ome,” (i.e., bacterial presence in buffers and other reagents) (Grahn et al., 2003; van der Horst et al., 2013; Olomu et al., 2020). Interestingly, the profile observed in negative controls and GF→GF mice was very similar, further validating the absence of endogenous bacteria in the GF group (Figure 6B). *Lactobacillus*, *Proteus*, and *Staphylococcus* were predominant across GF→CC and CC→CC samples. In contrast, *Bacteroides* and *Enterobacteriaceae* were the contaminants that dominated in negative control and GF→GF samples.

Principal coordinate analysis of Bray Curtis distances was used to evaluate differences at the level of bacterial community composition (beta diversity). PCoA plots show that GF→CC and CC→CC samples cluster together but separately from negative control and GF→GF samples, suggesting that bacterial communities are similar in composition in the microbiota harboring groups (Figure 6C). Nonetheless, PERMANOVA found a significant difference between the GF→CC and CC→CC samples (*p* = 0.03). ANCOM was used to test for individual species that differed significantly in abundance between the GF→CC and CC→CC groups. Remarkably, just one species was identified: *Lactobacillus reuteri* (*W* = 32; *U* = 45, *p* = 0.003) was present in half of the GF→CC samples and absent in all CC→CC samples (Figure 6D). We also note that although CC→CC and sham CC→CC offspring overall had similar bacterial composition, ANCOM revealed that the sham CC→CC group had more *Proteus* (*W* = 12; also captured in Figure 6B). However, this comparison did not quite reach significance in a non-parametric *t*-test (*U* = 19, *p* = 0.054).

**FIGURE 7:**
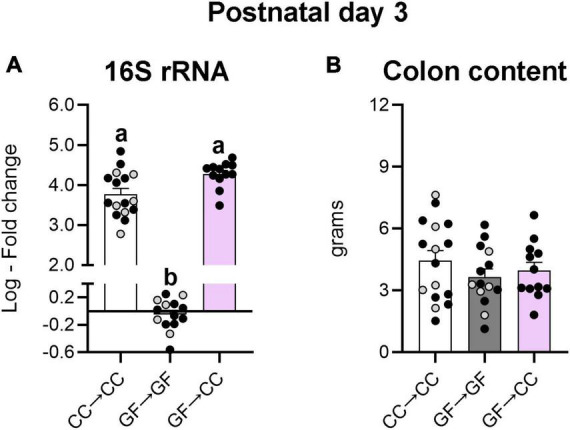
Introduction to a microbiota at birth normalizes the bacterial load of mice gestated germ-free at P3. **(A)** Relative quantification of the 16S rRNA gene from colon content showed similar levels of bacterial DNA in the groups harboring microbiota. GF→GF group was used as reference group for fold change calculations. **(B)** There were no differences in weight of the colonic content between groups. Group means with different letters are significantly different from each other. Mean + SEM and individual data points are depicted, with gray symbols representing sham cross-fostered mice in control groups.

**FIGURE 8:**
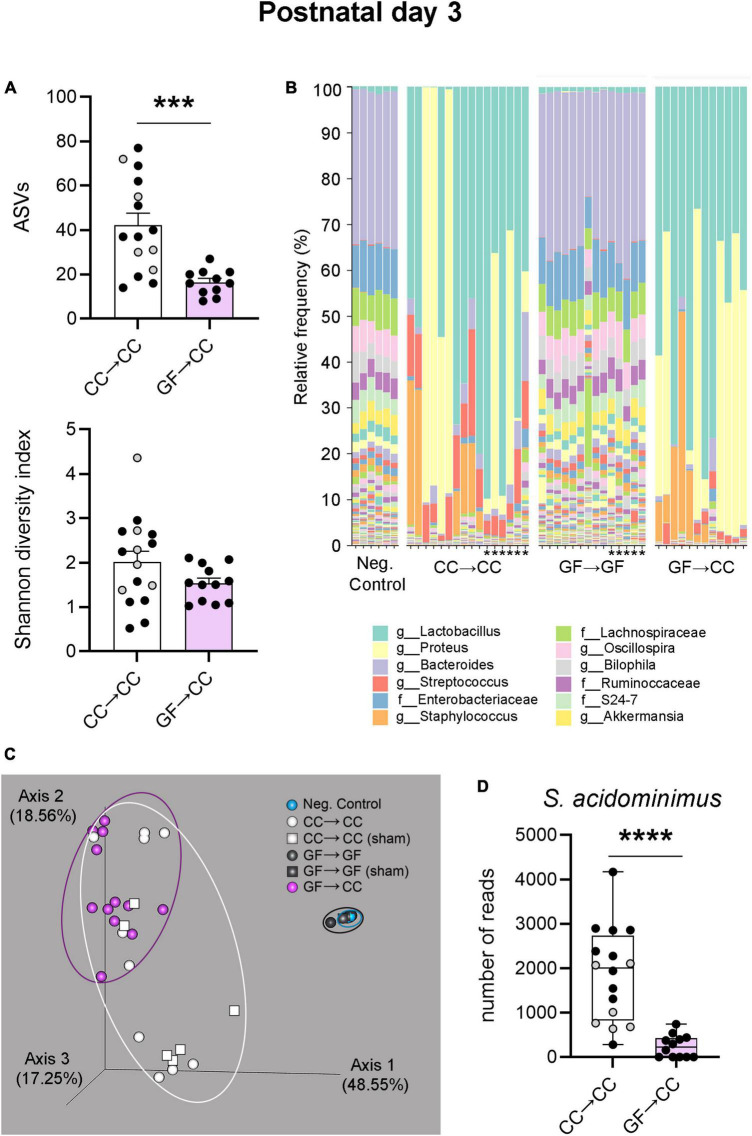
Introduction to a microbiota at birth largely normalizes bacterial composition of mice gestated germ-free by P3. **(A)** Measures of alpha-diversity revealed a difference between CC→CC and GF→CC groups in the number (richness) of ASVs: the CC→CC group showed doubled the number of ASVs (top). In contrast, when richness and abundance were considered by using the Shannon diversity index, there was no difference between groups (bottom). Mean + SEM and individual data points are depicted, with gray symbols representing sham cross-fostered mice in control groups. ***p = 0.0004. **(B)** Relative abundance of bacterial groups per sample (columns), showing that overall bacterial composition was similar between GF→CC and CC→CC groups, with the exception of higher abundance of *Streptococcus* in the CC→CC group. These two groups, however, were markedly different from negative and GF→GF groups. Asterisks indicate the sham cross-fostered mice in control groups. The 12 most abundant taxa are shown in the color key. Sequences were classified to the lowest taxonomic level they could confidently be identified. f, family; g, genus. **(C)** PCoA plots based on Bray-Curtis dissimilarity, showing that GF→CC and CC→CC individual samples (symbols) clustered somewhat further apart than at P7 but markedly separate from controls (clustering indicated with ellipses). Note that most samples for negative control and GF→GF groups overlap due to tight clustering; *n* = 6 and 14 in those groups, respectively. Percent of variance explained by principal coordinates is indicated on the axes. **(D)** Boxplots of the number of reads per sample of the ASV identified as *Streptococcus acidominimus*. Gray symbols represent sham, cross-fostering in control mice. ****p < 0.0001.

Thus, bacterial load and composition were largely identical between GF→CC and CC→CC mice at P7, but brain measures were not. Colon contents that were collected at P3 allowed us to test how quickly bacterial normalization occurs. Similar to what was seen at P7, bacterial load and colon content size did not differ between GF→CC and CC→CC groups at P3 (Figures 7A, B). However, colon contents of the CC→CC group had double the number of ASVs (*U* = 18, *p* = 0.0004) (Figure 8A, top), but similar values of the Shannon diversity index compared to the GF→CC group (Figure 8A, bottom). Taxon abundance assessment revealed that overall CC→CC and GF→CC were similar (and, again, vastly different from or negative controls of GF→GF samples), although *Streptococcus* appeared more predominant in CC→CC colons (Figure 8B). CC→CC and GF→CC groups at P3 clustered slightly further apart on PCoA plots than they did at P7 and PERMANOVA confirmed this difference (*p* < 0.002) (Figure 8C). However, ANCOM analysis again found only a single species that was significantly different in abundance between the groups: *Streptococcus acidominimus* [*W* = 44; *t*(26) = 5.18, *p* < 0.0001] was more predominant in the colons of CC→CC mice than in GF→CC mice at P3 (Figure 8D). In addition, we did not observe taxa abundance differences between CC→CC and sham CC→CC offspring at P3. Thus, when exposed to a normal microbiota on the day of birth, the neonatal gut microbiota was largely similar whether pups were gestated and born CC or GF, with some subtle differences, especially at the earlier timepoint (P3).

## 4. Discussion

We previously identified effects of the microbiota on microglia and neuronal cell death within hours after birth (Castillo-Ruiz et al., 2018a). In this study, a cross-fostering approach allowed us to test whether these effects are caused solely by the postnatal microbiota, or whether *in utero* exposure to the maternal microbiota plays a role. Overall, we find that the GF phenotype persists during the first postnatal week, despite successful acquisition of a microbiota at birth, suggesting a role for prenatal programming.

### 4.1. Microglia, cell death, and gross development effects

Microglial colonization of the brain and neuronal cell death are two of the most prominent neurodevelopmental events during the newborn period in mice. The number of microglia increases rapidly after birth and microglia undergo major morphological and gene expression changes during this period (Dalmau et al., 2003; Schwarz et al., 2012; Crain et al., 2013; Lai et al., 2013; Sharaf et al., 2013; Christensen et al., 2014; Matcovitch-Natan et al., 2016; Castillo-Ruiz et al., 2022). Similarly, developmental neuronal cell death is concentrated during the first postnatal week in mice (Ahern et al., 2013; Mosley et al., 2017). Microglia are quite sensitive to the microbiota. Erny et al. (2015) demonstrated increased microglial labeling in adult GF mice, and extended that to mice in which the microbiota was severely depleted in adulthood with antibiotics or which lacked a complex microbiome by virtue of being colonized by only three bacterial species. These findings suggest continuous regulation of microglia by the microbiome throughout life.

In the CA1 oriens and S1 we found that mice born GF, regardless of microbial status at P7, had more microglia than CC mice, suggesting persistence of the GF microglial phenotype in the GF→CC group. The ARC had a similar microglia pattern but we were underpowered to detect an effect. In contrast, in the PVN we observed partial normalization of the GF phenotype by the cross-fostering manipulation. The PVN is enriched in blood supply in comparison to neighboring regions (van den Pol, 1982), and this pattern develops during the first days postnatal in rats and mice (Menendez and Alvarez-Uria, 1987; Frahm et al., 2012). We speculate that microbial metabolites may be more accessible to the PVN via its nascent rich blood supply than to the other brain regions examined here. Consistent with this hypothesis, administration of bacterial metabolites to adult GF mice can rescue microglial numbers, morphology, and physiology (Erny et al., 2015, 2021). Moreover, gut-derived bacterial metabolites cross the blood-brain barrier *in vivo* (Frost et al., 2014) and influence microglia function *in vitro* (Erny et al., 2021).

For cell death, we found an effect of group in the ARC, with greater cell death in both GF→CC and GF→GF than in CC→CC mice. Interestingly, the ARC is involved in food intake, which is increased in GF mice (Bäckhed et al., 2004). In the CA1 oriens and PVN we did not find an effect of group on cell death, probably due to the fact that this process has tapered off in these regions by P7 (Ahern et al., 2013; Mosley et al., 2017). We did not assess the phenotype of the cells undergoing cell death in this study, however, they are likely to be mainly neurons based on previous reports in the neonatal brain (Zuloaga et al., 2011) and the neuron-like morphology shown by the cells we quantified.

Gross development was also affected by prenatal microbial absence, with GF→CC and GF→GF mice having greater forebrain size and body weight than CC→CC mice at P7. These measures may be dependent on mouse strain or diet, as they are found in some studies of GF mice but not others (Bäckhed et al., 2004; Fleissner et al., 2010; Khosravi et al., 2015; Selwyn et al., 2015; Kawase et al., 2017; Castillo-Ruiz et al., 2018a; Vuong et al., 2020). It is notable that most of the GF effects that we identified previously on microglia, cell death, and gross development in Swiss Webster mice at P0 and P3 (Castillo-Ruiz et al., 2018a) were replicated here at P7. Therefore, the GF phenotype persists throughout at least the first postnatal week. Because the brain undergoes extensive development during this time (Reemst et al., 2016), our past and current results could help explain why exposing GF rodents to microbes beyond the early postnatal window does not normalize some brain and behavior measures (Sudo et al., 2004; Clarke et al., 2013; Desbonnet et al., 2014). Similarly, introduction to a wild/more diverse mouse microbiota protects against diet-induced obesity if introduced to CC mice on P2, but not if the introduction is delayed to P15 (Hild et al., 2021).

As mentioned above, the co-housing of GF mice with CC mice at birth reduces microglial numbers in comparison to GF mice when examined in adulthood. Our results suggest that the normalization of brain measures is not immediate. Similarly, delayed effects of microbiota colonization have been reported in adult mouse colon (El Aidy et al., 2012; Johansson et al., 2015). Because microglia participate in diverse neurodevelopmental processes, including the phagocytosis of dying cells, neuro/gliogenesis, and synaptic pruning (Ferrer et al., 1990; Caldero et al., 2009; Paolicelli et al., 2011; Schafer et al., 2012; Cunningham et al., 2013; Shigemoto-Mogami et al., 2014; Lenz and Nelson, 2018), any deviations from their typical state could have significant effects on brain development.

### 4.2. Bacterial load and composition

Bacterial load and composition were largely identical between GF→CC and CC→CC mice at P7, suggesting rapid colonization of the gut in mice gestated GF and introduced to a microbiota at birth. In agreement, El Aidy et al. (2012) conventionalized adult GF mice and found that bacterial copy number reached its maximum after just 1 day. Overall, the species diversity observed in our study concurs with a previous report in neonatal mice showing low diversity at the end of the first week postnatal followed by a more stable and diverse community by weaning age (Pantoja-Feliciano et al., 2013). The predominant genera we observed are also in agreement with Pantoja-Feliciano et al. (2013), with dominance of *Lactobacillus* and *Streptococcus* during the first week postnatal. The prevalence of *Lactobacillus* may in part be due to its role inhibiting the growth of other bacterial communities via production of lactic acid from milk (Vandenbergh, 1993; Brownlie et al., 2022).

There were slight differences in alpha and beta diversity between CC→CC and GF→CC groups at P7 and ANCOM found a significant difference in one taxon: *L. reuteri* was greater in GF→CC than in CC→CC neonates. This finding is interesting given that administration of *L. reuteri* in its biofilm state normalizes microglia numbers in a mouse model of neonatal necrotizing enterocolitis (Wang et al., 2021). Therefore, it is tempting to speculate that *L. reuteri* may participate in the partial normalization of microglia seen in the PVN of GF→CC mice. However, *L. reuteri* was present in only half of the GF→CC mice at P7 and the presence of this species within the GF→CC group did not correlate significantly with microglial or cell death measures (not shown). We cannot rule out an association, however, as we may not have been sufficiently powered, especially for microglial measurements.

The GF→CC and CC→CC groups were already very similar in bacterial load and composition at P3. Nonetheless, we detected more pronounced differences between the groups at this age than at P7. The most notable was the predominance of *Streptococcus* in the CC→CC group, and as per the ANCOM results, this may in part relate to higher abundance of *S. acidominimus*. Interestingly, this species is sensitive to perinatal manipulations as shown by reduction in its numbers in the P2 mouse colon upon prenatal maternal stress (Jasarevic et al., 2018).

Overall, we did not find differences between true and sham cross-fostered mice for any of the variables assessed, with the exception of higher *Proteus* at P7 in the sham CC→CC group. Differences in gut microbiota composition due to cross-fostering were recently reported by Morais et al. (2020) in weanling and adult mice. However, in that study all non-cross-fostered pups remained undisturbed with the birth mother. Here, both sham and true cross-fostered pups experienced maternal separation and a disinfection regime, which may have more nearly equalized stress of the procedure across groups.

### 4.3. Does the maternal microbiota program brain effects?

The similarities between GF→CC and CC→CC bacterial communities are not surprising, given that the fetus develops in a sterile (or nearly sterile) womb and CC and GF offspring are expected to be on equal footing with respect to direct exposure to intestinal bacteria throughout gestation. Although we did not assess the maternal gut microbiota in our study, this microbiota was likely transferred promptly to GF→CC newborns via feces in the cage and foster dam behaviors: licking and grooming of pups after engaging in self-anogenital grooming and coprophagy. If so, our current results suggest that the maternal microbiota has programming effects on brain development *in utero*. Similarly *in utero* effects of the maternal microbiota have been reported for microglia and other neurodevelopmental events, including axonogenesis and sympathetic nervous system development (Thion et al., 2018; Kimura et al., 2020; Vuong et al., 2020). In fact, Thion et al. (2018) reported higher microglial numbers in GF mice as early as E14. We previously observed no differences in microglia and cell death between GF and CC mice in the hours just before birth (Castillo-Ruiz et al., 2018a) but this discrepancy may be due to the inflammation that occurs around time of parturition and that extends to the brain (Castillo-Ruiz et al., 2018b,^2022^).

Alternatively, it is possible that our cross-fostering manipulation (GF→CC) did not fully mimic the vertical transmission of microbes that occurs at birth in CC animals, and that the subtle differences we found in the microbiota could explain the persistence of the GF phenotype for most brain measures. There are at least two ways that the initial colonization of pups gestated and born GF versus CC may differ. First, mice born to a GF dam are not exposed to a vaginal microbiota during parturition. However, the maternal gut microbiota most powerfully shapes the newborn’s gut microbiota, and most maternal vaginal microbes are only very transiently found in the neonate’s gut (Sakwinska et al., 2017; Ferretti et al., 2018; Jasarevic et al., 2021). Nonetheless, the transient presence of vaginally-derived species could alter subsequent stages of gut colonization and affect development (Jasarevic et al., 2021). The two species identified as significantly different between GF→CC and CC→CC mice in our study: *S. acidominimus* at P3 and *L. reuteri* at P7, inhabit the gut but also may be found in the vagina (Smith and Sherman, 1939; Rabe et al., 1988; Tannock, 1995; Oh et al., 2010; Leccese Terraf et al., 2016). Thus, it is plausible that initial inoculation by vaginal microbes could account for differences in the abundance of these species, although this explanation is difficult to reconcile with the *greater* presence of *L. reuteri* in GF→CC mice.

A second possible reason that colonization during the first 7 days postnatal might not be identical in GF→CC and CC→CC newborns is *in utero* effects of the maternal microbiota on development of the fetal intestine or immune system. If arriving bacteria encounter a different environment in the GF→CC versus CC→CC colon, this could affect the persistence of specific species. Indeed, the maternal microbiota *in utero* plays a role in the development of the immune system (Gomez de Agüero et al., 2016). Our results suggest that if there are differences, they have only subtle effects on colonization since bacterial load and composition were remarkably similar in the GF→CC and CC→CC groups.

Finally, we cannot ignore the possibility that the persistence of the GF phenotype in the GF→CC group may be related to differences in microbial populations that we did not assess (e.g., fungi, viruses, or protozoans). However, at least for microglia, bacterial normalization may be more important as bacterial metabolites rescue microglial effects in adult GF mice (Erny et al., 2015, 2021).

## 5. Conclusion

In sum, we find that brain effects of gestating GF persist during the first postnatal week, despite successful acquisition of a microbiota at birth. These findings argue for an important role of the maternal microbiota during fetal life on neonatal brain development. Because our results identify specific neurodevelopmental events that are sensitive to prenatal microbial exposure, this information could aid in interpretation of future studies that evaluate programming effects of microbiota on brain physiology and behavior. In addition, our work identifies two potential species: *L. reuteri* and *S. acidominimus*, to target in future experiments examining the role of specific bacteria in orchestrating neonatal brain development.

## Data availability statement

The original contributions presented in this study are included in the article/supplementary material, further inquiries can be directed to the corresponding author.

## Ethics statement

The animal study was reviewed and approved by the Georgia State University’s Institutional Animal Care and Use Committee.

## Author contributions

AC-R and NGF designed the experiments and wrote the manuscript. AC-R, AG, NMR, DNP, CJGD, and BC performed the experiments. AC-R, HS, CJGD, and BC analyzed data. All authors contributed to the article and approved the submitted version.
